# The Royal Netherlands Football Association (KNVB) relative age solutions project—part one: a call to action

**DOI:** 10.3389/fspor.2025.1546829

**Published:** 2025-04-10

**Authors:** Adam Leigh Kelly, Frederike Zwenk, David Mann, Jan Verbeek

**Affiliations:** ^1^ Research for Athlete and Youth Sport Development (RAYSD) Lab, Birmingham City University, Birmingham, United Kingdom; ^2^ Royal Netherlands Football Association (KNVB), Zeist, Netherlands; ^3^ Department of Human Movement Sciences, Vrije Universiteit Amsterdam, Amsterdam, Netherlands; ^4^ Department of Psychology, Faculty of Behavioural and Social Sciences, University of Groningen, Groningen, Netherlands

**Keywords:** relative age effects, talent identification, talent development, athlete development, youth soccer, youth football

## Abstract

**Introduction:**

Despite its widespread prevalence in youth soccer, there seems to be no widely implemented intervention to moderate or overcome Relative Age Effects (RAEs). The purpose of this study was a call to action for stakeholders to propose relative age solutions to the Royal Netherlands Football Association (KNVB).

**Methods:**

The call to action consisted of a standardised, open-access questionnaire that contained questions focussed on: (a) the mechanisms of the proposal, (b) hypothesised effects, and (c) reference to empirical findings.

**Results:**

Following the initial screening of 185 submissions, a total of 143 eligible proposals were included. Each proposal was categorised by two project members based on a taxonomy to classify different approaches designed to reduce RAEs by: (a) altering the behaviour of observers, (b) implementing rules when selecting teams, or (c) adjusting competition structures. From this, 13 lower-order independent solutions were categorised.

**Discussion:**

Interestingly, whilst no new suggestions outside the existing literature were proposed in any of the submissions, only two have been empirically tested in soccer. Overall, the results present a useful first step in identifying possible relative age solutions. Due to the number of proposed solutions and their anecdotal nature, the next step for the KNVB was to utilise the knowledge of experts in the field via an adapted e-Delphi study to identify the most effective and feasible solutions to implement in practice (Part Two).

## Introduction

Relative Age Effects (RAEs) are well known phenomenon in soccer. Many studies have highlighted how relatively older youth players (i.e., those born near the start of the selection cut-off date) are afforded a variety of advantages and are overrepresented in both participation (e.g., recreational/grassroots) and developmental (e.g., academies/talent pathways) settings. In comparison, relatively younger youth players (i.e., those born towards the end of the selection cut-off date) are often at a disadvantage and remain underrepresented across the sport [see (1) for an overview in soccer]. Such RAEs have been found in youth soccer across all four corners of the globe, ranging from the United States (2) to Italy (3) and China (4) to Brazil (5). Contextual factors such as age [e.g., U12 vs. U19; (6)], competition level [e.g., recreational/grassroots vs. academies/talent pathways; (7)], gender [i.e., boys vs. girls; (8)], competitive success [e.g., more vs. less league points accrued; (9)], nationality [e.g., Belgium vs. France; (10)], and playing position [e.g., goalkeeper vs. defender; (11)] have been shown to influence the extent to which RAEs exist in soccer, highlighting the complexity involved when identifying, selecting, and developing young players.

The possible mechanisms that explain how RAEs occur remain inconclusive and mainly hypothetical. Initial relative age research in youth sport assumed that an advanced maturity status was the major underlying cause [e.g., the “maturation-selection hypothesis”; (12)]. Contrastingly, however, Hancock and colleagues (13) theorised the “social agents model” to suggest how it is in fact key stakeholders, including players [i.e., Galatea effect; (14)], coaches [i.e., Pygmalion effect; (15)], and parents [i.e., Matthew effect; (16)], who are responsible for perpetuating RAEs. Thereafter, Wattie and colleagues (17) proposed a “constraints-based developmental systems model” to explain how a variety of factors are responsible for RAEs in sport, based on environmental (e.g., access to soccer provision), individual (e.g., physical characteristics), and task (e.g., playing position) constraints. More recently, Kelly and colleagues (18) used the “personal assets framework” to underscore possible developmental outcomes due to RAEs in the immediate (i.e., personal engagement in activities, appropriate settings and organisational structures, and quality social dynamics), short-term (i.e., competence, confidence, connection, and character), and long-term (i.e., performance, participation, and personal development) timescales. Despite these theoretical efforts, limited empirical studies are available to show the exact causes of RAEs in youth soccer.

In light of the (dis)advantages that arise due to RAEs, researchers have suggested various potential solutions to mitigate these effects [see (19) for a review]. For example, applying 9-month age groups (20), rotating cut-off dates (21), implementing into coach education (22), adopting age-ordered shirt numbering (23), avoiding early deselection (24), using selection quotas (25), applying corrective adjustments (26), grouping athletes using characteristics other than age (27), considering a flexible chronological approach [i.e., playing-up and playing-down; (28)], moving each individual up to their next birthdate group on their birthday [i.e., birthday-banding; (29)], delaying the selection process (30), estimating developmental birthdates (31), and setting the average age of a team to a predetermined maximum (32) have all been proposed throughout the youth sport literature. Taken together, the general consensus from researchers has emphasised the need to explore potential solutions to combat RAEs. Despite numerous proposals from researchers, however, the design, implementation, and evaluation of such solutions in youth soccer remains mostly absent from the literature (23, 31). In addition to the lack of research testing potential relative age solutions, there are no studies that have explored stakeholder perspectives to better understand if these proposals are viable in real-life settings (33).

Although being considered a global leader when it comes to youth soccer development [e.g., (34, 35)], the Royal Netherlands Football Association (KNVB) are also guilty when it comes to creating RAEs [e.g., (36, 37)]. Indeed, they have received critical attention from popular media for creating inequitable opportunities for young players as a result of RAEs, which remain present throughout their existing age group structures [e.g., (38–40)]. As an example, data from UEFA (41) showed that the relatively youngest Dutch players selected to play in the men's 2016 U17 and U19 European Championships received the lowest proportion of playing time. Adding to the complexities of such RAEs at youth levels, it is also important to consider its implications on the long-term progression of players into international and professional levels [e.g., (42–44)]. In the case of the Netherlands, it appears to have knock-on effects on both the men's senior national team (45) as well as the men's Eredivisie domestic teams (10). As such, the efficacy of the current age group policies used in the Netherlands youth soccer pathways may not only have short-term effects at youth levels, but also potentially limits the potential talent transitioning to senior levels in the long-term. Therefore, the purpose of this study was a “call to action” for youth soccer stakeholders (e.g., academics, coaches, parents, policy makers, practitioners) to put forward their proposals for potential solutions that could be implemented into youth soccer to mitigate RAEs in the Netherlands.

## Methods

The call to action consisted of a standardised online survey that enabled the collection of responses from participants via a publicly available link. The link was included in a news article that communicated the KNVB's efforts to assemble solutions to mitigate RAEs in youth soccer. The article and accompanying link remained pinned on the KNVB's homepage for the duration of the data collection period. In addition, the article was shared via the KNVB's social media (news)outlets, including Twitter (now called X) and LinkedIn. The online submission form was available for participants to submit their solution from March 15th, 2021, to May 1st, 2021. The registration form was designed with Microsoft Forms (Microsoft Corporation, USA) and consisted of three parts focussed on: (a) the mechanisms of the proposed solution for RAEs, (b) hypothesised mitigating effect(s) of the solution on RAEs, and (c) any reference to empirical findings if applicable.

First, participants were required to provide consent for their proposed solution to be shared and used within this project. Following consent, participants were asked to provide a maximum 250-word summary on their proposed solution. After they provided their summary, participants could indicate if they were aware of whether their solution had been implemented or trialled in other sports. In addition, they could indicate if they believed the following factors related to RAEs were most affected by their solution: (a) modification of cut-off dates, (b) selection decisions regarding players, (c) grouping players within teams, or (d) using corrective (mathematical) algorithms to control for relative age differences. Finally, participants were asked to provide a short description on their course of action, such as an exemplary research design to trial their proposed solution within the context of the Netherlands youth soccer. For this, participants had the option to share any (peer-reviewed) research materials, if available, with the project team after they had registered their solution. See Supplementary File 1 for the Microsoft Forms (Microsoft Corp., Redmond, WA, USA) survey used for this study. The project was endorsed by the KNVB and ethically approved by the Health, Education, and Life Sciences Faculty Academic Ethics Committee at Birmingham City University (ethics code #9524).

## Results

The online survey yielded 185 responses. Following the initial screening of each submission, 26 were excluded because participants did not consent to their solution being used in this project. A further 25 were excluded because they did not propose any solution for RAEs in the Netherlands youth soccer. This resulted in 134 eligible submissions, with eight responses proposing multiple solutions (two, *n* = 7; three, *n* = 1), creating a total of 143 proposed solutions. Data were extracted and copied into a specifically designed Microsoft Excel (Microsoft Corp., Redmond, WA, USA) sheet, which enabled the assessment of each submission. Two project members independently assessed and categorised each of the 143 proposed solutions based on the taxonomy provided by Mann (1). This taxonomy consists of three higher-order themes of different approaches designed to reduce RAEs when performance is assessed objectively, including: (a) altering the behaviour of observers (*n* = 19), (b) implementing rules when selecting teams (*n* = 46), and (c) adjusting competition structures (*n* = 78). From this, 13 lower-order independent solutions were categorised. The classification process resulted in an initial percent agreement of 0.9. Any disagreements about the inclusion of a solution within a certain category were discussed amongst all authors and resolved by final consensus.

**Figure 1:**
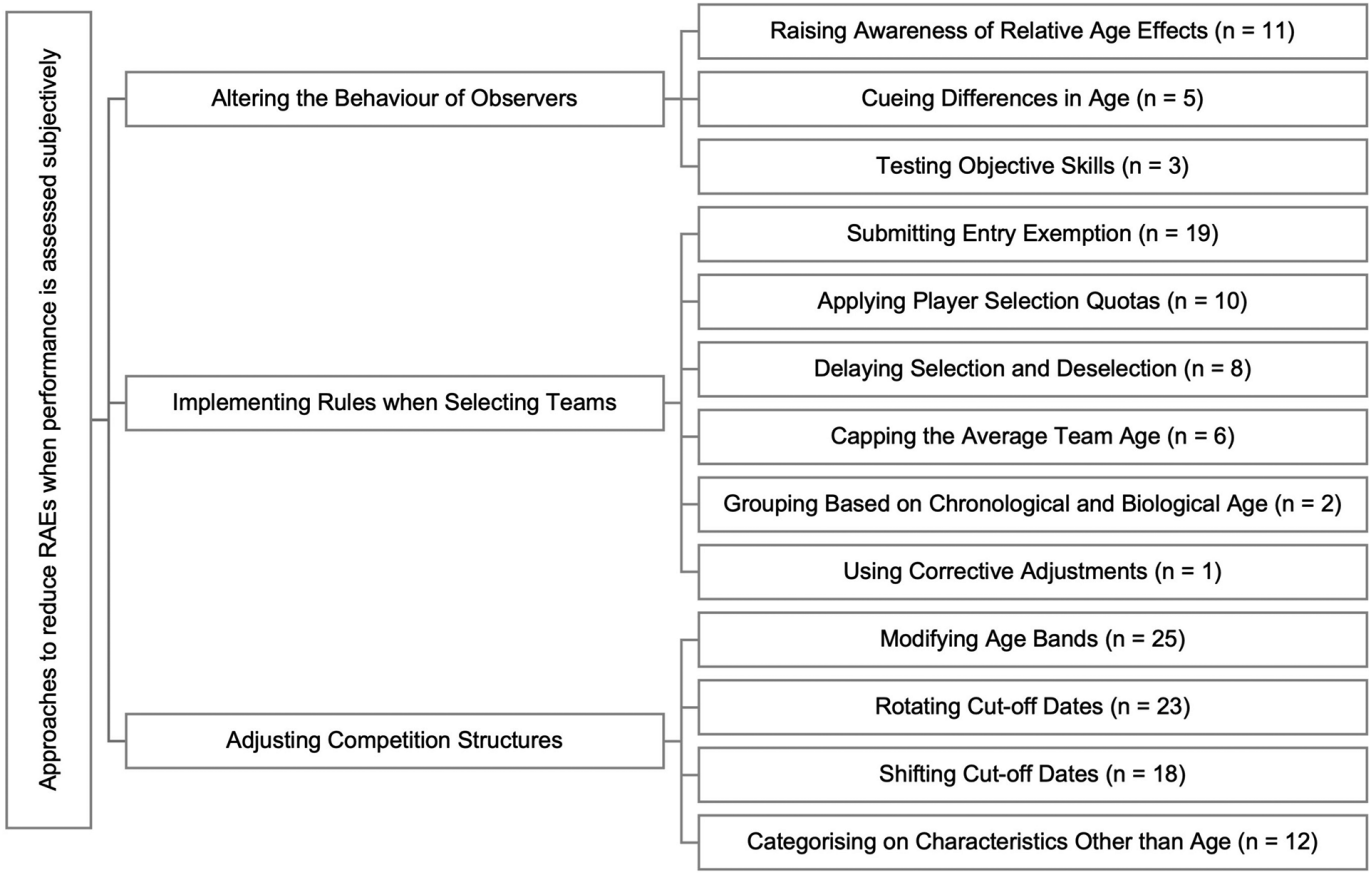
Categorisation of proposed solutions according to the taxonomy of Mann (1).

Overall, while no new suggestions outside the existing literature were proposed in any of the submissions, only two have been empirically tested in soccer previously (“cueing differences in age” and “categorising based on chronological and biological age”). The most frequent higher-order theme that was put forward by the participants was “adjusting competition structures”, with “modifying age bands” the lower-order theme that was suggested most often (Figure 1).

## Discussion

This discussion has been structured to consider each of the 13 lower-order independent solutions that were categorised based on the three higher-order themes: (a) altering the behaviour of observers (*n* = 3), (b) implementing rules when selecting teams (*n* = 6), and (c) adjusting competition structures (*n* = 4). Given no new solutions outside the current literature were proposed, we detail the existing research to help better understand the potential mechanisms of each possible solutions, as well as propose how each solution could potentially be adapted and implemented within Dutch youth soccer context.

### Altering the behaviour of observers

The first higher-order category considers solutions that alter the behaviour of observers (i.e., coaches, recruiters, talent scouts) when identifying and selecting young players. Overall, there were three solutions proposed in this category: (a) raising awareness of relative age effects (*n* = 11), (b) cueing differences in age (*n* = 5), and (c) testing objective skills (*n* = 3).

#### Raising awareness of relative age effects

National governing bodies and sport administrators are generally responsible for designing and delivering formal coach education programmes. The primary aim of such education provision is to ensure that coaches work effectively to create the most suitable learning environment for every young player to develop. Since the decision-making of coaches directly influences the development process of players (e.g., talent selection and/or identification), education programmes that raise awareness of RAEs (e.g., relative age information implemented into formal coaching awards) may be an effective method to reduce such effects (22, 47). Raising awareness of RAEs could be delivered in several ways [e.g., (48)]. Such approaches may include educating coaches on what RAEs are, outlining the causes and mechanisms of RAEs, and presenting examples of RAEs in action (47). Within the coach development pathway in Dutch soccer, there are several youth licencing requirements that all have their own educational programme. Here, the licencing process and accompanying education is regulated by the KNVB. As such, the implementation of new content focused on RAEs highly feasible.

Raising awareness could also change the way in which coaches think about long-term development. As an example, helping coaches understand how to remove the emphases on performance related outcomes (e.g., winning matches and leagues) and focusing on developmental assets (e.g., competence, confidence, connection, and character) could encourage long-term outcomes (e.g., performance, participation, and personal development) (49). Models such as the personal assets framework have been used to explain how RAEs affect the three dynamic elements (i.e., personal engagement in activities, appropriate settings and organisational structures, and quality social dynamics), which interact in the immediate timescale for sport development to occur (18, 50). For instance, the emphases on performance related outcomes might hinder the personal engagement of relatively younger players as they may lack confidence or advanced competencies. Therefore, educating practitioners and policy makers to change the emphasis of performance related outcomes to long-term developmental outcomes would likely affect how the dynamic factors interact, and subsequently enhance the sporting experience of relatively younger players.

#### Cueing differences in age

Identifying, selecting, and developing young players is traditionally performed by coaches. Although it is common for coaches to acquire players' birthdates during these processes, RAEs still occur. One reason why the awareness of a player's birthdate may not reduce RAEs is that it is not usually transformed into an explicit prompt or integrated into coaches decision-making processes. In order to facilitate this, it is suggested that each player's relative age is explicitly provided to those who are charged with making decisions during talent identification and selection procedures. Interestingly, to our knowledge, this proposal is only one of two studies that has been empirically tested in the literature (23, 31).

Specifically, Mann and van Ginneken (23) showed how cuing differences in relative age, via age-ordered shirt numbering, positively impacted coaches and selectors decision making. In this case, coaches and selectors are provided with information about the relative age of players based on the numbers (e.g., birth quarter 1–4 or birth month 1–12) on bibs that correspond to the relative age of the players. Not only does this explicitly show the relative age of the players, but also simultaneously aligns only to those who are on the field during training and competition. The age-ordered shirt numbering experiment of Mann and van Ginneken (23) was conducted with scouts who were required to rank the potential of players after viewing one match. In Dutch youth soccer context, such one-off selection evaluations are relatively uncommon. More typically, especially in grassroots soccer, players are assessed over multiple occasions during training and matches across the season. This would require that players wear the same shirt number during all these assessments, which might limit the apparent simplicity of this solution.

#### Testing objective skills

The final proposed solution to mitigate RAEs by altering the behaviour of observers is to include objective skill tests as part of evaluation and selection procedures. These tests, which could minimize the impact of physical capacity, might provide a more equitable context for relatively younger players to perform and compete. Examples of such measures includes tests for technical skill (e.g., skills testing, performance analysis), tactical ability (e.g., positional awareness, perceptual-cognitive expertise), and psychosocial characteristics (e.g., friendships, leadership skills). In addition, these objective tests that are suggested to measure relevant skills in the context of soccer might provide a better indication of future performance, although the validity of such performance tests are currently debated (51). Since the typical evaluation and selection procedures in Dutch youth soccer consist of coaches or scouts evaluating a player's future potential during several training sessions and matches, combining these assessments with objective tests results would require a significant change. In addition, nationwide skills testing would require many resources (e.g., financial investment, highly trained staff, time allocation), likely making it only possible for a limited number of attributes and timepoints.

### Implementing rules when selecting teams

The second higher-order category considers approaches that explicitly forces adjustments to grouping polices by implementing rules. There were six solutions proposed in this category: (a) submitting entry exemption (*n* = 19), (b) applying player selection quotas (*n* = 10), (c) delaying selection and deselection (*n* = 8), (d) capping the average team age (*n* = 6), (e) grouping based on chronological and biological age (*n* = 2), and (f) using corrective adjustments (*n* = 1).

#### Submitting entry exemption

Traditional age grouping and subsequent fixed cut-off dates means there is an upper age limit. As such, youth soccer players move up each year and remain in their respective under-age category until the end of the season (e.g., U11, U12, U13, etc.). This eligibility system allows younger players to compete in advanced age levels designed for older children, which is commonly known as “playing-up”. Kelly et al. (52) found an overrepresentation of early birth quartiles who play-up and suggested that playing-up may impact chronological age grouping twofold: (a) moderate RAEs by presenting a new cohort of later birth quartiles, and (b) create an “underdog effect” for relatively older players. Goldman et al. (53) also captured players' perceptions of playing-up and the ways in which it may impact their development, with findings involving a balance between: (a) challenge, and (b) progress.

In contrast, allowing relatively younger players to “play-down” (i.e., submitting entry exemption) may be another way in which RAEs could be mitigated. Indeed, “playing-down” an age group may offer a more suitable developmental setting for relatively younger players whilst they “catch-up” with their relatively older peers (54). However, in contrast to playing-up, older players are not ordinarily allowed to compete in competitions designed for younger age groups. This solution suggests that enabling players to delay their entry into a higher age category might overcome the associated disadvantages of being relatively younger, whilst also providing a more challenging environment for those in a younger age group. In this sense, the player is provided with additional support in terms of extra time for development across a more appropriate level of challenge. Examples of such eligibility delays are known in North American university sport systems as “redshirting” or “taking a victory lap of high school” (55).

In some cases, within Dutch youth soccer, players are allowed to compete in a younger age group; although, this exemption is currently only available for players aged 13–17 years who experience severely delayed maturation, making it uncommon in practice. This policy, however, could be adapted to meet the needs of relatively younger children (e.g., those born in the second half of the selection year can play down an age group; clubs are allowed up to two players to play-down). However, it is important to note that this proposed solution is yet to examined and could create unintended consequences through the possible stigma attached to “playing-down”, thus terminology such as “playing across” age groups may be more beneficial.

#### Applying player selection quotas

In traditional annual age grouping, all players born within a one-year period are eligible to play together in the same team. As a result, there is only one criterion when grouping players in a certain under-age team: all players must be born within that 12-month period (e.g., 1st January to 31st December). One proposed solution to mitigate RAEs was mandating (i.e., setting a quota) an equal proportion of players to be selected from each birth quartile (25%) or from the first and second half of the selection year (50%) [e.g., (56)]. This would ensure an even distribution of relatively older and younger players within teams, and thereby overcome the asymmetrical distribution of birthdates due to RAEs that favour relatively older players and disadvantaging relatively younger players (12).

Interestingly, selection quotas have been a part of a national discourse in an attempt to achieve racial transformation and inclusive representation in South Africa. For instance, Cricket South Africa mandated the “inclusion of a minimum average of six players of colour in the national team, of which at least two must be black Africans” (57). Such selection quotas, however, are yet to be evaluated in the context of RAEs. One potential reason could be the administrative burden that accompanies implementing this solution. In Dutch soccer, clubs can independently determine the structure of squads and thus implementing such quotas would significantly affect the autonomy of this process, while simultaneously, the KNVB has to check all squads during matchdays. To reduce these administrative challenges, the Belgian FA has recently announced that, for the time being, quotas will only apply to their elite academy teams (58).

### Delaying selection and deselection

In popular sports such as soccer, children are grouped not only on their chronological age (e.g., U9, U10, U11, etc.), but also based on their ability (e.g., representative and non-representative teams). This means that, within annual age groups, there are parallel teams and/or competitive levels for players of different abilities. Generally, those who are considered more talented are selected for representative teams (i.e., academies/talent pathways), providing them with access to higher quality coaching, better facilities, and additional resources. Because selection procedures based on a child's ability are biased to favour relatively older players, a potential solution to overcome RAEs is to delay the process of selection and deselection (24). Encouraging stakeholders to avoid deselection at young ages will allow individuals to remain exposed to practice, competition, and resources without the option or fear of being deselected (59). This could decrease the emphasis on short-term outcomes (i.e., performance, winning) as well as the accompanying stress and pressure, particularly during critical stages of development (e.g., decrease in performance, maturation, and/or injury), and instead increase a focus to develop all players in the soccer system for as long as possible.

An example of how such a delay in selection and deselection may look in practice is to implement specific regulations that ensure clubs remove ability groupings until maturation is achieved (12, 60). Not only would this allow any maturational differences to have disappeared or levelled out between players before selection occurs, but it would also ensure the impact of relative age differences are minimised due to increasing age. For instance, a relatively older U5 could have lived up to 20% longer than their relatively younger equivalents, whereas a relatively older U15 could only be up to 6.6% older than their relatively younger peers. Selecting closer to the intended outcome (i.e., expertise in adulthood) will also help with the accuracy of talent identification in football (61). For example, the Belgian FA implemented national youth teams specifically focused on later developing players. Specifically, these “Future Teams” provide players with the opportunity to play competitive football while remaining in the national talent pathway, with other member associations, including the Netherlands replicating this initiative (62).

#### Capping the average team age

An alternative to using a single cut-off date and possibly minimize RAEs is grouping players according to a maximum average team age. This Average Team Age (ATA) grouping procedure sets the average age of a team to a predetermined maximum and simultaneously determines the maximum age difference between the oldest and the youngest player on a team. As such, both the mean and the range of ages are defined on a team eligibility basis rather than on an individual eligibility basis, allowing any individual player to participate across several different age groups (63). Accordingly, the ATA grouping procedure can be devised as follows: “a competing squad shall consist of no more than ‘X’ players whose average age on the competition start date shall be no more than ‘Y’. No player in the team shall be more than ‘Z’ years older than the youngest player in the squad” (32, p. 114).

There is anecdotal evidence from Stoke City Football Club Academy in England who have trialled the ATA approach by mixing their U9 to U12 age groups. According to their academy staff, the grouping policy showed positive developmental opportunities for players in terms of social cohesion and additional challenges. Moreover, they stated how older players took on leadership roles when playing with younger academy teammates (64). Whilst this approach seems promising, it is yet to be empirically studied. In Dutch youth soccer, there is a long-standing tradition of (bi-)annual birth year grouping, therefore, implementing a new grouping approach based on a maximum average age would require significant changes to this process, especially at grassroots level. Similar grouping approaches, however, have been adopted in wheelchair rugby. Specifically, during a match at any given time, any four players may be on the floor if they do not collectively exceed eight points (with each player allocated a set number of points based on their functional mobility) (32). Indeed, the approach used here may help shed light on policy making and practical implementation of capping the average team age in the context of youth soccer.

#### Grouping based on chronological and biological age

A traditional underlying principle for annual age grouping in youth soccer is that children of a similar age will be of similar sizes and abilities and, as such, it will provide developmentally appropriate settings for all learners. Over the last two decades, however, a growing pool of literature has showed maturation status does not necessarily correspond to the chronological age of players (65), and thus maturity-associated differences might contribute to RAEs. Indeed, research has showed that there can be up to five years difference in biological age between those in the same chronological age, which can lead to later maturing players being systematically left out of academies, particularly those who are relatively younger (66). For instance, Hill et al. (67) showed that not one single late maturing player was part of their U15 and U16 English academy cohort, making it hard to consider how they can develop within the existing chronological structures.

To mediate the inequalities resulting from these maturity-related and relative age differences, it was proposed to group players based on their respective chronological birthdate *and* developmental birthdate (31). This approach estimates the developmental birthdate based on growth curves for stature, where the stature of a player is mapped on the average growth curve. In other words, stature is used as a proxy for biological maturation. For example, a 9-year-old Dutch boy would play in the U10. However, the child has the stature of 131 cm, which corresponds to a developmental age of 7 years and 10 months based on the normal growth curve of the Dutch male population. Grouping players is then based on the midway point of their chronological and developmental birth date. In this example, that would be 8 years and 5 months, reallocating the player to a lower age category (i.e., U9). Hence, this method could re-allocate players to a different age category if their estimated developmental birth date does not correspond with their chronological age. An empirical study by Helsen et al. (31) suggests that reallocation into new teams reduced both the variation in maturation status and the overrepresentation of players born in a certain birth quarter (i.e., RAEs). While including developmental birthdate based on stature would require some amendments to the traditional birthyear grouping, it could be implemented across the Dutch youth soccer context. For instance, stature is regularly assessed during primary and secondary school health checks and the outcomes can be used in tools such as the Dutch TNO Growth Predictor, to attain the corresponding chronological age based on the normal growth curve of the Dutch population (68).

#### Using corrective adjustments

In some sport contexts (e.g., long-jump, weightlifting, and swimming), performance can be measured objectively (i.e., in centimetres, grams, or seconds, respectively). Although this is not straightforward in team sports such as soccer, objective skills tests or performance analysis tools have been developed in which performance outcomes are assessed individually. For such measures, it is possible to adjust an individual's performance based on their relative age so that all children within the same chronological age group can be compared equally. Such corrective adjustments are hypothesised to mitigate RAEs because they create a more equitable comparison of performance across players with different relative ages (69). More specifically, performance outcomes in terms of distance, weight, or time can be age-corrected for relatively younger players, creating a more equitable context to evaluate players. In swimming, for example, Cobley et al. (26) showed that when correctively adjusted swim times based on longitudinal reference data were implemented from accurate estimates of the relationship between decimal age (i.e., chronological and relative), RAEs were predominantly absent across age-group and selection levels. Thus, such an approach using performance metrics in soccer (e.g., player match analysis data) may be an avenue for future research for Dutch youth soccer.

### Adjusting competition structures

The third and final higher-order category considers solutions that require adjustments to the current competition structure in youth soccer. There were four solutions proposed in this category: (a) modifying age bands (*n* = 25), (b) rotating cut-off dates (*n* = 23), (c) shifting cut-off dates (*n* = 18), and (d) categorising on characteristics other than age (*n* = 12).

#### Modifying Age bands

Age grouping in youth soccer generally applies 24- or 12-month bands. This means that players born in the first month can be up to 23- or 11-months older than the youngest player in an age band, respectively. Given that these age differences lead to RAEs, it is suggested that modifying the age band may help solve RAEs. To be specific, modifying age bands could result in decreasing the maximum relative age difference, whereby using multiple within-year cut-off dates limits the maximum age difference to, for instance, 6- or even 3-months. Moreover, as suggested by the so-called Novem system by Boucher and Halliwell (20), a smaller banding of 9-month age categories breaks the annual 12-month structure, reducing the systematic advantage of players born shortly after the cut-off date. This also creates greater diverse perspectives and a non-linear development pathway, since players become the relatively youngest and oldest throughout their development due to the 9-month annual cycles.

#### Rotating cut-off dates

January 1st is the most common cut-off date to group players based on their chronological age, although this can vary depending on each countries own policies (70). This cut-off date is then maintained throughout all youth age categories until senior soccer, creating a relative age difference that provides a consistent advantage for those children born earlier in the selection year throughout their entire development. Consequently, solutions to mitigate RAEs target this age advantage so that this advantage of being the oldest is balanced over all players within the selection year. Accordingly, rotating cut-off dates has been proposed as a potential solution for RAEs.

In practice, there are several ways that the cut-off date can be rotated. For example, every season the cut-off date could be shifted three months from January 1st to April 1st, which would see the oldest players move up to the older age cohort to become the youngest players amongst that cohort [i.e., the Relative Age Fair (RAF) cycle; (21, 71)]. Another version of this proposal that is used in the England Squash Talent Pathway is termed “birthday-banding” (29), whereby young athletes move up to the next age-group on their birthday. In Dutch youth soccer, for example, when a ‘U13' player turns 14, they would advance to the ‘U14’ age group and remain in that age group until their next birthday. The aforementioned “modifying age bands” suggestions that aim to break the structure based on a multiple of twelve, such as 9-, 15-, or 21-month grouping procedures, would also create a new cut-off date after every annual cycle (72).

#### Shifting cut-off dates

Since 1997, most international youth soccer competitions adopt the cut-off date of January 1st (73). Although this shift has not resulted in any changes regarding the prevalence of RAEs in youth soccer, also not in the Netherlands, there have been some interesting cases where alignment of the cut-off date at school and in sport suggests a higher likelihood of RAEs. For instance, cut-off dates that are the same date for school enrolment and sport are likely to (dis)advantage the same children in both domains, while shifting cut-off dates would distribute these (dis)advantages across different domains (i.e., school and sports), so that each child could benefit from being relatively older and younger in diverse contexts (74). As such, shifting cut-off dates to deviate away from the educational cut-off date (e.g., September 1st) and other sports has been proposed as a possible solution for RAEs in soccer. However, after the US Soccer Federation changed its birth-year registration cut-off date from August 1st to January 1st (in 2015) to align the US youth soccer calendar with international standards, while simultaneously provide clearer information on player birthdates to “lessen” RAEs, it only shifted the athletes experience relative age (dis)advantages. Moreover, the related outcomes were negatively perceived by stakeholders at various levels of the sport (75). Therefore, the Dutch FA should be cautious about using this approach as it could lead to unintended consequences.

#### Categorising on characteristics other than age

To ensure equitable competition for players, individuals are grouped according to their chronological age. It is expected that players of a comparable same age will be of similar ability, size, and physique. However, individual variation in physical characteristics and the maturation status of players is not accounted for in chronological age grouping (13). As such, relatively older players, who may be more likely to have a physical advantage, are subsequently considered more gifted by coaches who misconstrue the enhanced growth, physical capabilities, and maturational status with talent (76, 77). Hence, alternative grouping approaches, such as “bio-banding”, have been proposed to mitigate RAEs. So far, no nationwide alternative grouping approaches have been implemented in the Netherlands, although anecdotal evidence suggests individual clubs might use bio-banding next to regular age grouping [e.g., (78)].

This grouping approach accounts for individual variability in physical characteristics by grouping players on their biological age (i.e., maturity status), often using their percentage of predicted adult height [see (79) for a review]. As such, same-aged peers might play on different teams according to their maturational status in contrast to using chronological age as a grouping criterion. According to players, a key advantage of this grouping approach is that early-maturing athletes, who tend to be relatively older, can “play-up” thereby gaining valuable learning experiences from having to compete with older players (80). However, it is essential to note that biological age and relative age are two independent constructs, whilst bio-banding has been specifically designed to moderate maturity-related biases and has yet to be tested on its influence on RAEs.

### Limitations

It is important to recognise that this call for action was made via the KNVB, and thus the benefits and drawbacks of these proposed solutions could differ based on other national contexts (81). For instance, the Netherlands is a relatively small but densely populated country where soccer is the most popular sport, which could have impacted on the knowledge of stakeholders, the mechanisms of the proposals, and their subsequent benefits and drawbacks. In contrast, the call to action was publicly available and not limited to Dutch participants, which could have yielded wider suggestions that may not be applicable to the Netherlands. Although, in our opinion, this widened the pool of proposed solutions rather than hindered the methods. In addition, RAEs effect boys and girls differently [e.g., (2, 8)], therefore potential solutions may benefit (or not benefit) each gender in different ways [see (82) for a review], which was generally not acknowledged throughout the proposed solutions. Overall, we recommend the reader should reflect on how effective and feasible the proposed solutions are within their respective youth soccer environment before testing them in practice.

## Conclusion

Relative Age Effects are well established in youth soccer. To date, however, limited attempts have been made to better understand potential solutions in real-life youth soccer settings. In this call to action, 13 proposed solutions were suggested to mitigate RAEs, although no new solutions outside the existing literature were presented. Whilst the purpose of this study was to underscore possible relative age solutions, it was beyond its scope to explore the potential benefits and drawbacks of each proposal. Importantly, though, it should be recognised how solutions that alter the behaviour of observers and require adjusting competition structures can be driven from the KNVB as the governing body of Dutch youth soccer. Whereas, solutions that implement rules for selecting teams are much less dependent on a nationwide rollout and can more easily be introduced at regional/local club levels. A crucial next step for this research group was to utilise the knowledge of experts in this field via an adapted e-Delphi study, in order to identify the most effective and feasible solutions to apply in practice (Part Two). Furthermore, it will be important to design, implement, and evaluate solutions that are perceived to be the most effective and feasible across a variety of Dutch youth soccer settings.

## Data Availability

The original contributions presented in the study are included in the article/Supplementary Material, further inquiries can be directed to the corresponding author.
