# Supplementary material for: The Royal Netherlands Football Association (KNVB) relative age solutions project—part one: a call to action
Source: Front Sports Act Living. 2025 Apr 10;7:1546829. doi: 10.3389/fspor.2025.1546829 (PMC12018424; doi:10.3389/fspor.2025.1546829)
Supplement: Supplementary file 1 [file Datasheet1.pdf]

The Royal Netherlands Football Association (KNVB) Relative Age Solutions Project—Part One: A Call for Action

Supplementary file

Online survey

Introduction

The relative age effect is a complex phenomenon across sports and entails the over-representation of earlier born athletes compared to their later born peers. Based on this advantage in age, it is thought that it provides youth athletes with a competitive advantage as well. Many studies to date have investigated the relative age effect across domains, it's supposed causal mechanisms and consequences for youth athlete development.

Despite its widespread prevalence, to date, there seems to be no widely implemented intervention to solve or moderate the relative age effect. As such, the Royal Netherlands Football Association provides this call to action. Specifically, we are reaching out to researchers and practitioners to share their ideas on how they think we can solve (or moderate) RAEs. Moreover, the Royal Netherlands Football Association not only would like to collect new and innovative ideas but work together with researchers and practitioners on actual testing of these solutions in youth football the coming years.

Forget yet another study stumbling on the relative age effect, it is time for solutions!

Please circle as appropriate

Do you understand that the Royal Netherlands Football Association may contact you to ask for more details regarding your proposed solution?

❍ Yes ❍ No

Do you understand that by participating in this study, the Royal Netherlands Football Association may use your proposed solution for further investigation regarding solving the relative age effect?[1]

❍ Yes ❍ No

Do you understand that participation in this study is entirely voluntary?

❍ Yes ❍ No

Age verification: I am over 18 years old.

❍ Yes ❍ No

Name?

____

Country of residence?

____

What is your e-mail address?

____

Please provide in 250 words a summary of the proposed solution.

____

Has your proposed solution ever been applied to sports?

❍ Yes, team sports

❍ Yes, individual sports

❍ Both

❍ Other

❍ No

In the case of ‘Other’, where has your proposed solution been applied to then?

Which of the following factors does your proposed solution target (the most)?

- ❍ Cut-off date
- ❍ Grouping strategies
- ❍ Selection policy
- ❍ Algorithm
- ❍ Other, please specify …

Has your solution been previously been published elsewhere or is it based on material from other sources (e.g., peer-reviewed studies)?

- ❍ Yes
- ❍ No

In the case of ‘Yes’, could you refer to the original source?

____

Please describe in a maximum of 250 words how your proposed solution would affect player development?

____

What is needed to test your potential solution in a real-world experimental design?

____

Do you have any additional material to support your proposed solution such as graphs, stats or figures? Please upload it right here.

[1] If that is the case, wherever possible we will contact you and discuss further steps. In the outputs from the project, your proposed solution will not be identifiable in any way to you.
